# Novel homozygous stop-gain pathogenic variant of *PPP1R13L* gene leads to arrhythmogenic cardiomyopathy

**DOI:** 10.1186/s12872-022-02802-7

**Published:** 2022-08-06

**Authors:** Samira Kalayinia, Mohammad Mahdavi, Golnaz Houshmand, Mahshid Hesami, Maryam Pourirahim, Majid Maleki

**Affiliations:** 1 Cardiogenetic Research Center, Rajaie Cardiovascular Medical and Research Center, Iran University of Medical Sciences, Tehran, Iran; 2 Rajaie Cardiovascular Medical and Research Center, Iran University of Medical Sciences, Tehran, Iran

**Keywords:** *PPP1R13L* gene, Dilated cardiomyopathy, Arrhythmogenic cardiomyopathy, Genetic, Variant, Whole-exome sequencing

## Abstract

**Background:**

Arrhythmogenic cardiomyopathy (ACM) is a heritable cardiac disease with two main features: electric instability and myocardial fibro-fatty replacement. There is no defined treatment except for preventing arrhythmias and sudden death. Detecting causative mutations helps identify the disease pathogenesis and family members at risk. We used whole-exome sequencing to determine a genetic explanation for an ACM-positive patient from a consanguineous family.

**Methods:**

After clinical analysis, cardiac magnetic resonance, and pathology, WES was performed on a two-year-old ACM proband. Variant confirmation and segregation of available pedigree members were performed by PCR and Sanger sequencing. The *PPP1R13L* gene was also analyzed for possible causative variants and their hitherto reported conditions.

**Results:**

We found a novel homozygous stop-gain pathogenic variant, c.580C > T: p.Gln194Ter, in the *PPP1R13L* gene, which was confirmed and segregated by PCR and Sanger sequencing. This variant was not reported in any databases.

**Conclusions:**

WES is valuable for the identification of novel candidate genes. To our knowledge, this research is the first report of the *PPP1R13L* c.580C > T variant. The *PPP1R13L* variant was associated with ACM as confirmed by cardiac magnetic resonance and pathology. Our findings indicate that *PPP1R13L* should be included in ACM genetic testing to improve the identification of at-risk family members and the diagnostic yield.

**Supplementary Information:**

The online version contains supplementary material available at 10.1186/s12872-022-02802-7.

## Introduction

Arrhythmogenic cardiomyopathy (ACM) is a rare cardiomyopathy with 1 per 5000 prevalence and is defined by ventricular enlargement, myocardial fibro-fatty replacement, conduction defects, inflammation, and apoptosis, leading to ventricular arrhythmias [1]. Arrhythmogenic right ventricular dysplasia (ARVD) and subsequently ACM have been an evolving diagnosis throughout the recent decade with cardiac magnetic resonance imaging (CMRI) integration into the phenotype identification of cardiomyopathies [2]. A major cause of heart failure, ACM can occur due to genetic factors. Genetic ACM is more severe, has an earlier onset, and is heterogeneous; it is caused by mutations in genes encoding cardiac desmosomes, namely cadherins, plakins, and armadillo proteins [3–5]. More than 60% of ACM cases have desmosomal gene mutations; however, non-desmosomal gene mutations have also been detected [3, 6, 7]. A genetic variant interpreted as a pathogenic variant fulfills a major criterion set by the Task Force Criteria (TFC) for ACM recognition [8]. ACM can be typically present in an autosomal dominant pattern with incomplete penetrance and variable expression, but autosomal recessive inheritance is also known in a small number of cases [9, 10]. Many ACM patients have no identifiable mutation, with only one-fifth of all ACM cases familial. These patients may have pathogenic variants or causative genes not yet identified. Recently, a meta-analysis indicated that family history among ACM patients with non-desmosomal gene mutations had a lower frequency [11]. This clear gap in our understanding of ACM has led to continuous surveys to identify other causative genes of ACM.

*PPP1R13L* is a novel causative gene that encodes the inhibitor of apoptosis-stimulating p53 protein (iASPP), which plays regulatory and inflammatory roles in desmosomes. *PPP1R13L* is located on chromosome 19q13.32 [12, 13]. In 2009, Simpson and colleagues identified a frameshift variant (p.Ser322GlnfsTer4) in 13 animals with cardiomyopathy and woolly haircoat syndrome. They indicated that the bovine PPP1R13L sequence had 91.9% sequence similarity and 88.7% identity with the human PPP1R13L sequence [14]. Falik-Zaccai and coworkers identified a *PPP1R13L* missense variant (p.Tyr747Ter) in 3 patients with DCM associated with mild skin, teeth, and hair abnormalities (cardio-cutaneous syndrome) [15]. In 2019, in a study by Poloni and colleagues, a novel missense variant (p.Ala620Pro) of *PPP1R13L* was recognized in an ACM patient [13]. Recently, Robinson and coworkers reported three frameshifts, one nonsense, and one stop-loss in five pediatric dilated cardiomyopathy (DCM)-affected families. These studies provide evidence that *PPP1R13L* is a causative gene in cardiomyopathies (Table 1). Genetic investigation of patients with cardiomyopathy is significant for possible personalized cure and risk prediction of family members [16]. Recently, cardiomyopathy genetic research has been improved by the emergence of next-generation sequencing (NGS) approaches. Here, we identified a homozygous stop-gain pathogenic variant of *PPP1R13L* in a DCM/ACM Iranian patient by whole-exome sequencing [17].

**Table 1:** The reported variants in *PPP1R13L* and their related phenotypes

No	Nucleotide change	Amino acid change	dbSNP	CADD	SIFT	Polyphen-2	PROVEAN	Mutation taster	ClinVar	ACMG	Condition	References
1	c.2241C > G	p.Tyr747Ter	rs1114167453	43	–	–	–	D	P	P	DCM,CCS	[15, 20]
2	c.1610delG	p.Pro537LeufsTer100	–	–	DMG	PD	D	PD	LP	P	DCM	[20]
3	c.2486_2487delinsCT	p.Ter829Serext*2	–	–	–	–	–	–	–	VUS/P	DCM	[20]
4	c.736_764del	p.Pro246GlyfsTer15	–	–	DMG	PD	N	PD	P	P	DCM	[20]
5	c.2167A > C	p.Thr723Pro	–	31	DMG	PD	D	D	VUS	VUS/LP	DCM	[20]
6	c.1537delC	p.Val513CysfsTer124	rs34338233	28.5	DMG	PD	D	PD	LP	P	DCM	[20]
7	c.1219C > T	p.Gln407Ter	rs1290915929	34	–	–	–	D	LP	P	DCM	[20]
8	c.2396G > C	p.Trp799Ser	rs748300482	29.7	DMG	PD	D	D	VUS	VUS/LP	DCM	[20]
9	c.956_962dup	p.Ser322GlnfsTer4	–	–	DMG	PD	N	PD	–	P	CWH	[14]
10	c.1858G > C	p.Ala620Pro	rs774027921	23.8	DMG	PD	N	D	–	VUS	ACM	[13]

P: pathogenic; LP: likely pathogenic; D: disease causing; VUS: uncertain significance; DMG: damaging; T: tolerated; N: neutral; PD: probably damaging; DCM: dilated cardiomyopathy; CCS: cardio-cutaneous syndrome; CWH: cardiomyopathy and woolly haircoat syndrome; ACM: arrhythmogenic cardiomyopathy

## Methods

### Study subject and ethics statement

**Fig. 1:**
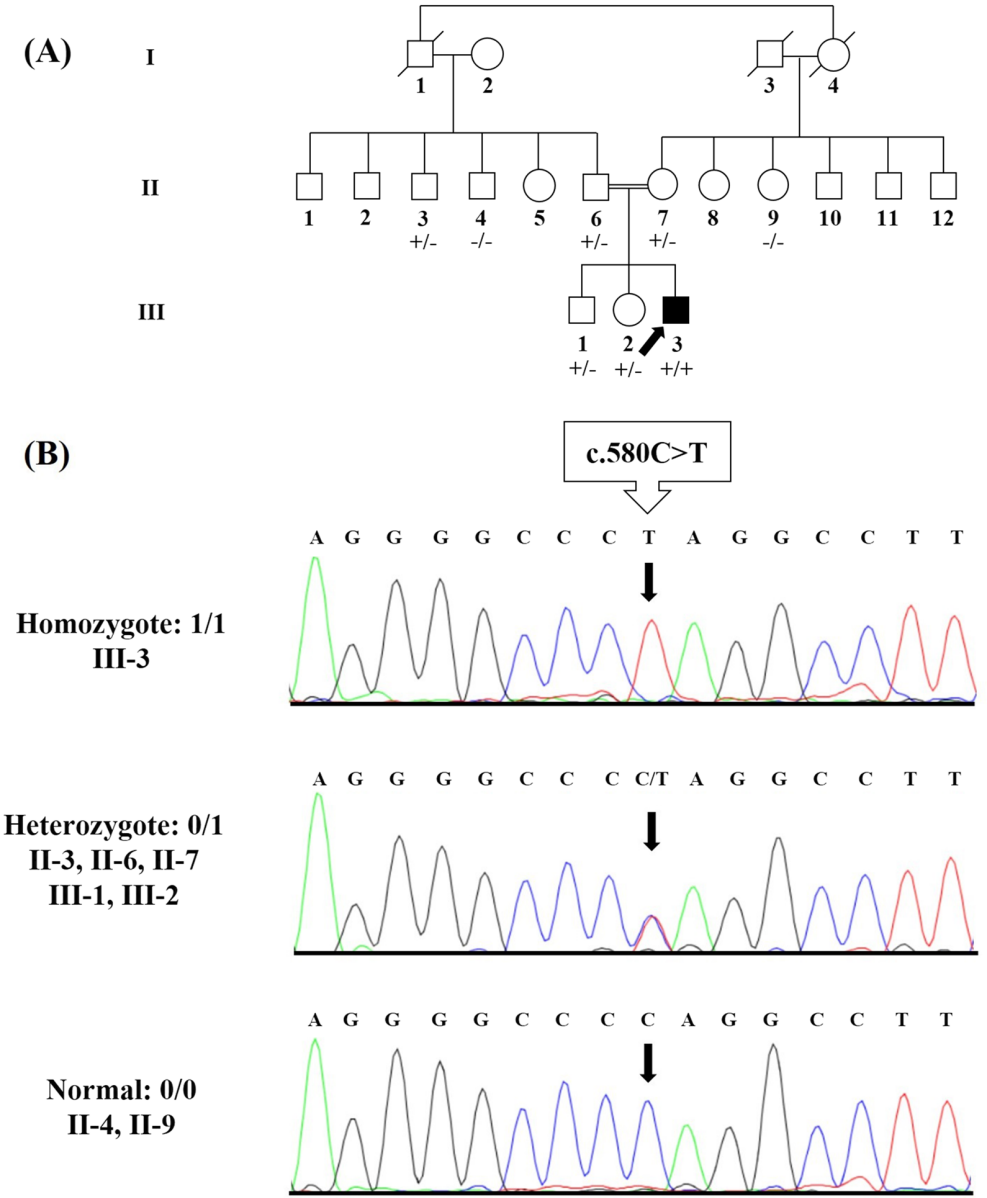
The image illustrates Family pedigree and genetic analysis of a *PPP1R13L* c.580C > T variant. **a** The pedigree of the family is shown herein. **b** The genotypes of a novel stop-gain pathogenic variant c.580C > T (p.Gln194Ter) were detected in the affected proband as homozygous and in his siblings and parents as heterozygous. The other available individuals had a normal sequence or carried the variant as heterozygous

In this study, a consanguineous Iranian family with three offspring, namely a two-year-old ACM son, a thirteen-year-old healthy son, and a ten-year-old healthy daughter, was referred to the Cardiogenetics Research Center, Rajaie Cardiovascular Medical and Research Center, Tehran, Iran (Fig. 1a). The affected offspring (III-3, Fig. 1a) was born via cesarean section. Based on clinical, laboratory and morphological findings, ACM was diagnosed when he was six months old, three to four weeks after the infant was generally unwell. There was no history of fever in his past medical history. Blood, urine, and metabolic parameters were normal. His growth was acceptable, and he had no other abnormalities up to two years old. Despite medical treatment, however, his ACM progressed to end-stage heart failure, and he underwent heart transplantation at the age of eighteen months. There was not skin or hair abnormalities in the proband. No family history was observed in the pedigree. All the medical histories and the clinical information were collected in Rajaie Cardiovascular Medical and Research Center, Tehran, Iran, in accordance with the Declaration of Helsinki, and the study protocol was approved by the institutional ethics committee (IR.RHC.REC.1399.044). Written informed consent was obtained from all participants.

### Cardiac magnetic resonance imaging and pathology

Cardiac magnetic resonance was conducted with a 1.5T MAGNETOM Avanto (Siemens Healthcare, Erlangen, Germany). The standard protocol was performed with the acquisition of steady-state free precession cine imaging in long- (four-, two-, and three-chamber) and short- (breath-hold) axis cine pictures plus the right ventricular inflow-outflow view. The short Tau inversion-recovery (STIR) sequence in long- (four-, two-, and three-chamber) and short- (breath-hold) axis cine pictures was taken. Early and late gadolinium enhancements of a short-axis stack of the three long-axis views and the right ventricular inflow-outflow images were collected by using magnitude and phase-sensitive inversion recovery reconstructions after the administration of 0.15 mmol/kg of gadoterate meglumine (gadolinium-DOTA, Dotarem, Guerbet SA, Paris, France).

In addition, the myocardium obtained from the transplanted heart was formalin-fixed and paraffin-embedded for analysis. Full-thickness sections from the right and left free ventricular walls and septa were prepared. The slides were stained with both hematoxylin and eosin (H&E) and Masson trichrome stains.

### Whole-exome sequencing and segregation analysis

Genomic DNAs of the family members were extracted from their peripheral blood by using the DNSol Midi kit (Roche: Product No. 50072012). The WES of the proband (III-3, Fig. 1a) was performed by Macrogen (Seoul, South Korea) on an Illumina HiSeq 4000. Raw data (FASTQ format) were analyzed in the Cardiogenetics Research Center, Rajaie Cardiovascular Medical and Research Center, Tehran, Iran. In general, according to our in‐house setup NGS analysis pipeline (i.e., mapping, variant calling, and annotation), point variants, duplications, and deletions/insertions (indels) were detected. Public international databases such as the 1000 Genomes Project (http://www.1000genomes.org/), the Genome Aggregation Database (gnomAD) (https://gnomad.broadinstitute.org/), the Greater Middle East (GME) (http://igm.ucsd.edu/gme/), and Iranome (http://www.iranome.ir) were used to analyze the minor allele frequency (MAF) of the variants. Reported mutations in ClinVar (https://www.ncbi.nlm.nih.gov/clinvar) and Human Gene Mutation Database (HGMD) (http://www.hgmd.cf.ac.uk/ac/index.php) as likely pathogenic/pathogenic were considered the priority. Bioinformatics analysis was carried out on the remaining variants through the application of online tools such as Combined Annotation-Dependent Depletion (CADD) (https://cadd.gs.washington.edu/home), SIFT (https://sift.bii.a-star.edu.sg/), PolyPhen-2 (http://genetics.bwh.harvard.edu/pph2/), PROVEAN (http://provean.jcvi.org/index.php), and MutationTaster (http://www.mutationtaster.org/) to predict the variant effect of the function/structure of the protein.

Further, ENTPRISE-X (http://cssb2.biology.gatech.edu/entprise-x/) was employed to predict the consensus of nonsense variants. The variants predicted by most of the tools as damaging were selected for segregation analysis. The standards of the American College of Medical Genetics and Genomics (ACMG) were applied for the interpretation of the variants [18].

One primer pair was designed by using Primer3 (v.04.0) (http://bioinfo.ut.ee/primer3-0.4.0/). Forward primer 5′-ACACCAACCCTTCCACTAATG-3′ and reverse primer 5′-GTCAGACTCGTTCCAGGCT -3′ were applied to amplify the variant sequence. Polymerase chain reaction (PCR) was performed on a SimpliAmp™ Thermal Cycler (Thermo Fisher Scientific) with 300 ng of genomic DNA, 1.5 mmol/L of MgCl2, 200 mmol/L of dNTP, 10 pmol/L of primers, and 1 U of Taq DNA polymerase (Amplicon, UK) at 95 °C for 5 min of incubation and amplification (30 s at 95 °C, 30 s at 60 °C, and 30 s at 72 °C; 35 cycles). The PCR products were sequenced on an ABI Sequencer 3500XL PE (Applied Biosystems), and the sequences were analyzed by CodonCode Aligner (v.7.1.2) (https://www.codoncode.com/aligner/).

## Results

### Clinical analysis

**Fig. 2:**
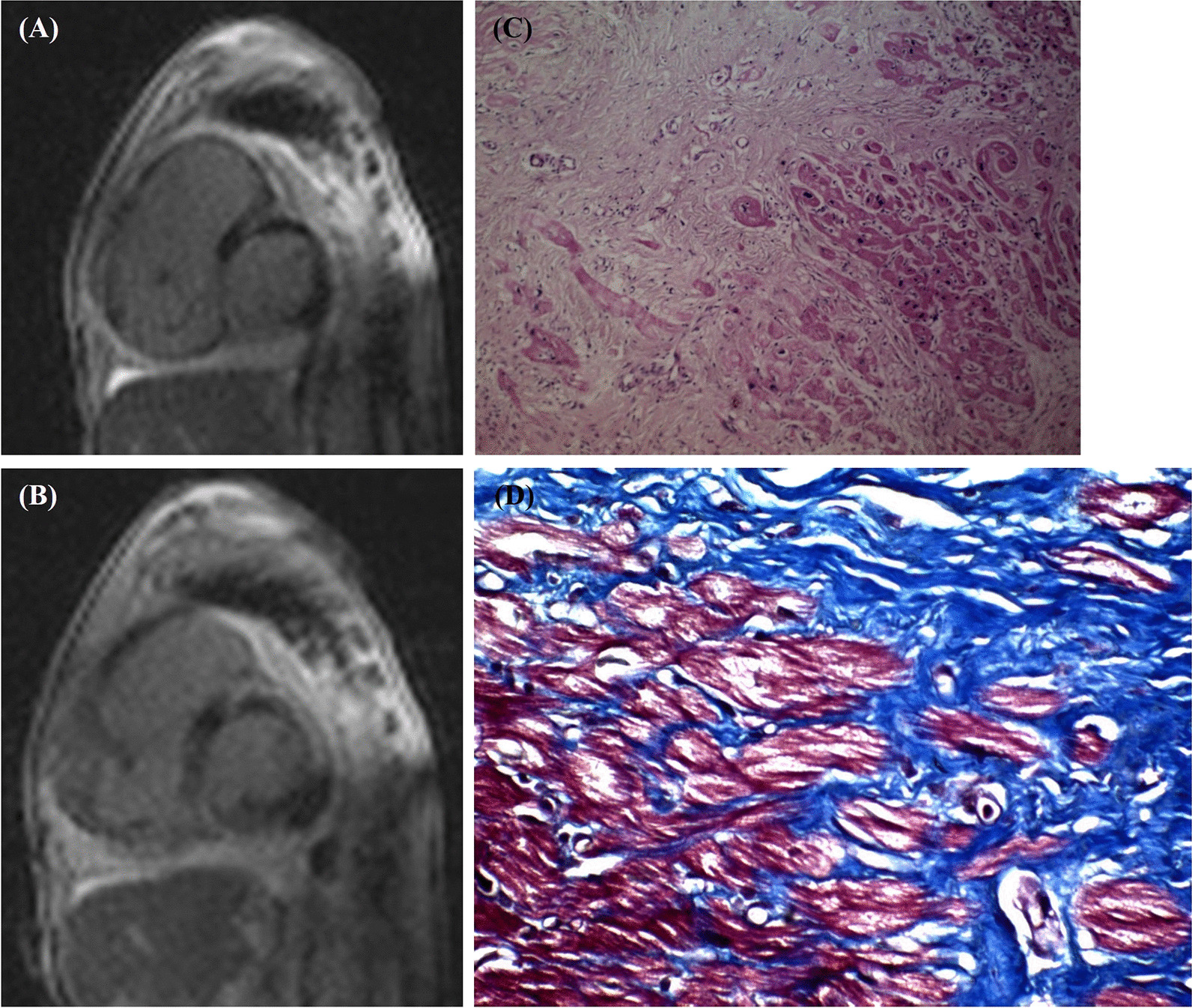
The image depicts dilated cardiomyopathy as confirmed by pathology and cardiac magnetic resonance. **a** and **b** The late enhancement sequences at the basal and mid-level short-axis views, respectively, show nearly-circumferential subepicardial enhancements in the basal-to-mid inferior, inferolateral, and mid-anterolateral walls of the left ventricle. **c** Microscopic examination shows cardiac myocytes with nuclear enlargement, anisonucleosis, and vacuolar degeneration (H&E X400). **d** Trichrome staining of the right ventricular free wall shows replacement fibrosis

Cardiac magnetic resonance showed a severely enlarged right ventricle with severely reduced right ventricular systolic function, concomitant with a mildly enlarged left ventricle with severely reduced left ventricular systolic function (Additional file 1). The right ventricular end-diastolic volume indexed to the body surface area was 265 mL/m^2^, and the right ventricular ejection fraction was 13%. The left ventricular end-diastolic volume indexed to the body surface area was 96 mL/m^2^, and the left ventricular ejection fraction was 19%. The right ventricular inflow-out flow cine images (Additional file 2) visualized segmental aneurysmal formations with dyskinesia in the right ventricular outflow tract and the sub-tricuspid region. The STIR sequences showed no inflammation or edema. Late gadolinium enhancement images demonstrated nearly-circumferential subepicardial fibrosis in the basal-to-mid inferior, inferolateral, and mid-anterolateral walls of the left ventricle (Fig. 2a, b). Given the cardiac magnetic resonance features of regional wall motion abnormalities in the right ventricle and biventricular systolic dysfunction and fibrosis pattern in the left ventricle, the phenotype was compatible with ACM with biventricular involvement. In addition, the 12-lead electrocardiograph (ECG) showed RS pattern in the inferior leads and QR pattern in V1 with poor R progression, and persistent prolonged S wave in the precordial leads. (Additional file 3). The clinical surveys of other available members of the pedigree were normal.

Macroscopic examination of the transplanted heart showed the enlargement of both ventricular cavities. The maximal thickness of the right and left free ventricular walls was 1 cm and 1.5 cm, respectively, and the interventricular septum had a maximal thickness of 1.4 cm. Histological features revealed nuclear enlargement, anisonucleosis, and vacuolar degeneration. Trichrome staining of the right and left ventricular free walls showed foci of interstitial and replacement fibrosis (Fig. 2c, d).

### Molecular analysis

WES detected a novel homozygous stop-gain pathogenic variant of *PPP1R13L* (NM_006663.4:exon4:c.580C > T: p.Gln194Ter) in the proband. This variant is predicted to make a truncated PPP1R13L protein and was not reported in any databases. The c.C580T:p.Q194X variant with a CADD Phred of 38 was predicted as disease-causing, deleterious, and pathogenic by MutationTaster, ENTPRISE-X, and ACMG, respectively.

### *PPP1R13L* genotype among the family members

In the confirmation and segregation analysis of the family members, the only affected individual, proband (III-3, Fig. 1b), had the c.C580T:p.Q194X variant as homozygous, and his siblings and parents had the variant as heterozygous (II-6, II-7, III-1, and III-2, Fig. 1b). The other available unaffected members of the pedigree had a normal sequence (II-4 and II-9, Fig. 1b) or carried the variant as heterozygous (II-3, Fig. 1b). Clinical examinations of all available members, heterozygous individuals, were also completely healthy.

## Discussion

ACM diagnosis has been evolved with the use of advanced cardiac imaging and electrophysiological studies [2, 19]. The phenotype of our case was determined by cardiac MRI features which are as follows: Dilated RV index versus left ventricle, presence of regional wall motion abnormalities in the right ventricle plus reduced left ventricular function and fibrosis pattern which all are more in favor of ARVD with concomitant LV involvement; ACM per definition versus DCM.

**Fig. 3:**
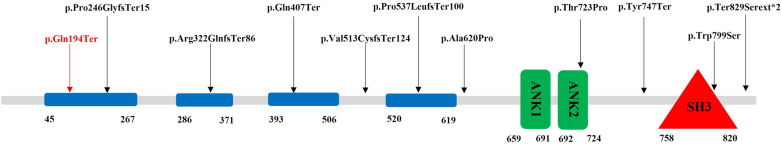
The image demonstrates the distributions of the reported variants of *PPP1R13L*. The regions and domains were obtained from UniProt (https://www.uniprot.org/)

Our study highlighted *PPP1R13L* as an important candidate in ACM. Given that the first symptom of ACM is mostly sudden death, early recognition of at-risk individuals is crucial. WES is a powerful approach to detect novel pathogenic variants of heart diseases. With the aid of this tool, we identified a novel *PPP1R13L* stop-gain variant (c.580C > T: p.Gln194Ter) in an autosomal recessive ACM pedigree. In recent years, *PPP1R13L* variants have been identified in patients with cardio-cutaneous syndrome [15, 20], cattle woolly haircoat syndrome [14], DCM [20], and ACM [13] (Table 1). The location of all reported variants was shown in Fig. 3. *PPP1R13L* encodes the iASPP protein in the intercalated discs of cardiomyocytes [21], and iASPP interacts with desmin and desmoplakin. Indeed, iASPP represses NF-κB transcriptional activity, which is followed by heart failure development [22]. A deficiency of iASPP has been recently related to autosomal recessive cardiomyopathy in Wa3 mice [23] and Poll Hereford calves [14]. Notari and colleagues identified iASPP as a gatekeeper of ACM and a desmosome key regulator. They formed a ternary assembled with desmin and desmoplakin and showed that deficient iASPP in mice led to ventricular tachycardia, arrhythmia, ACM, and sudden death [24]. In our study, the c.580C > T variant, in both *PPP1R13L* alleles, was predicted to cause truncated iASPP proteins. Djouadi and coworkers indicated the PPARγ pathway activation in iASPP-truncated hearts and its implication in ACM adipogenesis. The exact mechanism of the iASPP effect on the heart is unknown; nonetheless, it seems that deficient iASPP cannot inhibit p53 and its enhanced activity leads to the activation of PPARγ and elevated LPIN 1 levels [25]. The LPIN 1 protein is assembled with PPARγ for gene expression regulation in the fatty acid oxidation process [26]. Deficient iASPP protein does not interact with desmin and desmoplakin and, thus, may render individuals susceptible to ACM.

In light of the results of the present study, we suggest that *PPP1R13L* could be as a potential ACM candidate gene. Further functional studies are, however, needed to confirm the importance of this gene in ACM and clarify the exact mechanisms with a view to formulating new therapeutic approaches.

## Conclusions

In the era of NGS technologies, especially WES, there are opportunities to resurvey diseases with no known genetic reasons. Such approaches in recent years have revealed ACM-related variants in individuals with neither family history nor ACM symptoms. Our study revealed homozygous variant in the *PPP1R13L* gene as a cause of ACM. Thus, the best way to achieve the most accurate diagnosis is to combine clinical and genetic tests and interpret the results collectively.

## Accession Number

The accession number of the variant in ClinVar is as follows:

NM_006663.4 (PPP1R13L): c.580C > T (p.Gln194Ter): VCV001188825.1

## Supplementary Information

**Additional file 1**: The four-chamber view cine sequence showing enlarged RV size and severely reduced biventericular function plus the aneurysmal formation of the sub-tricuspid region

**Additional file 2**: The right ventricular inflow-outflow view cine sequence showing the aneurysmal formation of Right ventricular outflow and sub-tricuspid regions

**Additional file 3**: The image illustrates the electrocardiograph of the proband (III-3). 12-lead ECG showed peaked P wave and RS in the inferior leads and QR in V1 with poor R progression and persistent and prolonged S precordial leads

## Data Availability

All data generated or analyzed during this study are included in this published article.

## References

[1] Basso C, Corrado D, Marcus FI, Nava A, Thiene G (2009). Arrhythmogenic right ventricular cardiomyopathy. Lancet.

[2] Corrado D, Marra MP, Zorzi A, Beffagna G, Cipriani A, De Lazzari M, Migliore F, Pilichou K, Rampazzo A, Rigato I (2020). Diagnosis of arrhythmogenic cardiomyopathy: the Padua criteria. Int J Cardiol.

[3] Groeneweg J, van der Heijden J, Dooijes D, van Veen T, van Tintelen J, Hauer R (2014). Arrhythmogenic cardiomyopathy: diagnosis, genetic background, and risk management. Neth Hear J.

[4] James CA, Syrris P, Van Tintelen JP, Calkins H (2020). The role of genetics in cardiovascular disease: arrhythmogenic cardiomyopathy. Eur Heart J.

[5] Thiene G, Nava A, Corrado D, Rossi L, Pennelli N (1988). Right ventricular cardiomyopathy and sudden death in young people. N Engl J Med.

[6] Te Rijdt WP, Jongbloed JD, De Boer RA, Thiene G, Basso C, Van Den Berg MP, Van Tintelen JP (2014). Clinical utility gene card for: arrhythmogenic right ventricular cardiomyopathy (ARVC). Eur J Hum Genet.

[7] James CA, Jongbloed JD, Hershberger RE, Morales A, Judge DP, Syrris P, Pilichou K, Domingo AM, Murray B, Cadrin-Tourigny J (2021). International evidence based reappraisal of genes associated with arrhythmogenic right ventricular cardiomyopathy using the clinical genome resource framework. Circ Genomic Precis Med.

[8] Musunuru K, Hershberger RE, Day SM, Klinedinst NJ, Landstrom AP, Parikh VN, Prakash S, Semsarian C, Sturm AC (2020). Genetic testing for inherited cardiovascular diseases: a scientific statement from the American Heart Association. Circ Genomic Precis Med.

[9] Xu T, Yang Z, Vatta M, Rampazzo A, Beffagna G, Pillichou K, Scherer SE, Saffitz J, Kravitz J, Zareba W (2010). Compound and digenic heterozygosity contributes to arrhythmogenic right ventricular cardiomyopathy. J Am Coll Cardiol.

[10] Lorenzon A, Pilichou K, Rigato I, Vazza G, De Bortoli M, Calore M, Occhi G, Carturan E, Lazzarini E, Cason M (2015). Homozygous desmocollin-2 mutations and arrhythmogenic cardiomyopathy. Am J Cardiol.

[11] Xu Z, Zhu W, Wang C, Huang L, Zhou Q, Hu J, Cheng X, Hong K (2017). Genotype-phenotype relationship in patients with arrhythmogenic right ventricular cardiomyopathy caused by desmosomal gene mutations: a systematic review and meta-analysis. Sci Rep.

[12] Bergamaschi D, Samuels Y, O'Neil NJ, Trigiante G, Crook T, Hsieh J-K, O'Connor DJ, Zhong S, Campargue I, Tomlinson ML (2003). iASPP oncoprotein is a key inhibitor of p53 conserved from worm to human. Nat Genet.

[13] Poloni G, Calore M, Rigato I, Marras E, Minervini G, Mazzotti E, Lorenzon A, Mura IEAL, Telatin A, Zara I (2019). A targeted next-generation gene panel reveals a novel heterozygous nonsense variant in the TP63 gene in patients with arrhythmogenic cardiomyopathy. Heart Rhythm.

[14] Simpson M, Cook R, Solanki P, Patton M, Dennis J, Crosby A (2009). A mutation in NFκB interacting protein 1 causes cardiomyopathy and woolly haircoat syndrome of Poll Hereford cattle. Anim Genet.

[15] Falik-Zaccai TC, Barsheshet Y, Mandel H, Segev M, Lorber A, Gelberg S, Kalfon L, Ben Haroush S, Shalata A, Gelernter-Yaniv L (2017). Sequence variation in PPP 1R13L results in a novel form of cardio-cutaneous syndrome. EMBO Mol Med.

[16] McMurray JJ (2015). Improving outcomes in heart failure: a personal perspective. Eur Heart J.

[17] Rowczenio DM, Noor I, Gillmore JD, Lachmann HJ, Whelan C, Hawkins PN, Obici L, Westermark P, Grateau G, Wechalekar AD (2014). Online registry for mutations in hereditary amyloidosis including nomenclature recommendations. Hum Mutat.

[18] Richards S, Aziz N, Bale S, Bick D, Das S, Gastier-Foster J, Grody WW, Hegde M, Lyon E, Spector E (2015). Standards and guidelines for the interpretation of sequence variants: a joint consensus recommendation of the American College of Medical Genetics and Genomics and the Association for Molecular Pathology. Genet Med.

[19] Corrado D, Zorzi A, Cipriani A, Bauce B, Bariani R, Beffagna G, De Lazzari M, Migliore F, Pilichou K, Rampazzo A (2021). Evolving diagnostic criteria for arrhythmogenic cardiomyopathy. J Am Heart Assoc.

[20] Robinson H, Zaklyazminskaya E, Povolotskaya I, Surikova Y, Mallin L, Armstrong C, Mabin D, Benke P, Chrisant M, McDonald M (2020). Biallelic variants in PPP1R13L cause paediatric dilated cardiomyopathy. Clin Genet.

[21] Dedeić Z, Sutendra G, Hu Y, Chung K, Slee EA, White MJ, Zhou FY, Goldin RD, Ferguson DJ, McAndrew D (2018). Cell autonomous role of iASPP deficiency in causing cardiocutaneous disorders. Cell Death Differ.

[22] Gordon JW, Shaw JA, Kirshenbaum LA (2011). Multiple facets of NF-κB in the heart: to be or not to NF-κB. Circ Res.

[23] Herron BJ, Rao C, Liu S, Laprade L, Richardson JA, Olivieri E, Semsarian C, Millar SE, Stubbs L, Beier DR (2005). A mutation in NFkB interacting protein 1 results in cardiomyopathy and abnormal skin development in wa3 mice. Hum Mol Genet.

[24] Notari M, Hu Y, Sutendra G, Dedeić Z, Lu M, Dupays L, Yavari A, Carr CA, Zhong S, Opel A (2015). iASPP, a previously unidentified regulator of desmosomes, prevents arrhythmogenic right ventricular cardiomyopathy (ARVC)-induced sudden death. Proc Natl Acad Sci.

[25] Assaily W, Rubinger DA, Wheaton K, Lin Y, Ma W, Xuan W, Brown-Endres L, Tsuchihara K, Mak TW, Benchimol S (2011). ROS-mediated p53 induction of Lpin1 regulates fatty acid oxidation in response to nutritional stress. Mol Cell.

[26] Finck BN, Gropler MC, Chen Z, Leone TC, Croce MA, Harris TE, Lawrence JC, Kelly DP (2006). Lipin 1 is an inducible amplifier of the hepatic PGC-1α/PPARα regulatory pathway. Cell Metab.

