# Supplementary figures and images for: Novel homozygous stop-gain pathogenic variant of PPP1R13L gene leads to arrhythmogenic cardiomyopathy
Source: BMC Cardiovasc Disord. 2022 Aug 6;22:359. doi: 10.1186/s12872-022-02802-7 (PMC9356459; doi:10.1186/s12872-022-02802-7)

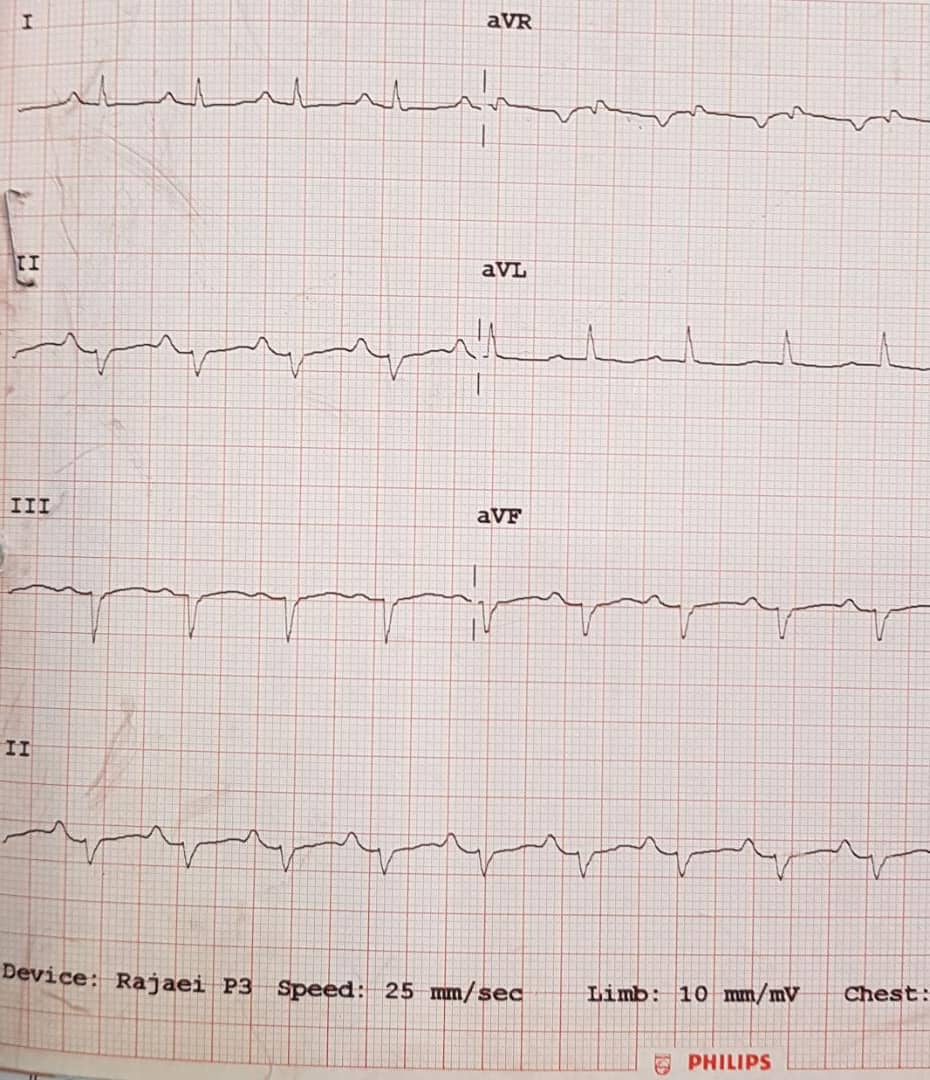

Supplement: Supplementary file 3 — Additional file 3: The image illustrates the electrocardiograph of the proband (III-3). 12-lead ECG showed peaked P wave and RS in the inferior leads and QR in V1 with poor R progression and persistent and prolonged S precordial leads [file 12872_2022_2802_MOESM3_ESM.jpg]
